# Charity Misconduct on Public Health Issues Impairs Willingness to Offer Help

**DOI:** 10.3390/ijerph182413039

**Published:** 2021-12-10

**Authors:** Lijun Yin, Ruzhen Mao, Zijun Ke

**Affiliations:** Guangdong Provincial Key Laboratory of Social Cognitive Neuroscience and Mental Health, Department of Psychology, Sun Yat-Sen University, Guangzhou 510006, China; maorzh@mail2.sysu.edu.cn (R.M.); keziyun@mail.sysu.edu.cn (Z.K.)

**Keywords:** charity, misconduct, news, help, public health

## Abstract

Charity organizations positively impact our societies but charity misconduct impairs people’s willingness to contribute to charity and functional health systems on public health issues. This study investigates the impact of charity misconduct on people’s willingness to offer help on public health issues and possible ways of reducing the negative impact brought by charity misconduct news through four studies (*N_total_* = 1269). Results showed that charity misconduct on public health issues significantly reduced individuals’ willingness to offer help via both the charity involved with the misconduct and any charity they prefer (Study 1 and 2). Furthermore, news on charity misconduct reduced people’s general willingness to help in contexts that did not involve charity (Study 3). Finally, presenting charity nonmisconduct news after charity misconduct news increases individuals’ willingness to offer help via the nonmisconduct charity (Study 4), suggesting a potential way to nudge people to provide help in the fight against the negative impact brought by charity misconduct news. The findings show the backfire of reporting charity misconduct news and have important implications for potential ways to facilitate people to offer help.

## 1. Introduction

Charity is established to help society and many researchers of charitable giving focus on how to increase donations and effective giving [1,2,3]. A significant proportion of nonprofit organizations are comprised of health care organizations [4]. Societies face ever-growing health challenges such as the COVID-19 pandemic outbreak that dramatically impacts individuals’ physical and psychological health [5], increasing demands on aid from nonprofit sectors. Although most charitable organizations carry out their duty properly, frauds in a single charity sector tarnish the reputation and future fund-raising capability of nonprofit organizations worldwide [6,7,8,9]. There is a particularly disproportionate incidence of fraud in health and human services [10]. Donors are sensitive to information about charitable organizations, such as aid impact and effectiveness [2]. Individuals’ dislike toward charities with low charity performance metrics would lead to more selfish decisions and less giving behaviors [11]. Reputation is one of the most important forces that drive charitable giving for non-profit organizations [12,13,14]. Charity misconduct might set back the recovering process from public health emergencies like the COVID-19 pandemic by influencing individuals’ willingness to offer help. The negative impact can further be amplified by mass media [15]. Knowing how news on charity misconduct influence individuals’ willingness to help is important for policymakers to effectively allocate public resources and for practitioners to develop efficient strategies to fight against potential negative impact. The current study focuses on the impact of charity misconduct news on individual willingness to offer help and potential ways of reducing negative effects.

Charity misconduct news conveys the message of a charity’s misconduct, including deceiving the donors. Individuals are sensitive to and have an aversion toward being deceived. As a common phenomenon [16], deception is morally unacceptable [17]. Direct victims of deception suffer substantial psychological distress besides financial distress [18] and would allocate less money to deceivers in a dictator game [19]. The experience of being deceived impairs individuals’ likeability toward liars [20], harms trust that cannot be fully recovered even after receiving apologies and observing trustworthy actions [21], and elicits higher activity in the anterior insula [17] that is associated with social emotions [22], disgust, aversion, negative arousal, and processing negative experiences [23,24,25,26].

Different from interpersonal deception in daily life that usually affects a small range of people, nonprofit fraud and misconduct are significant and costly [9,10]. The misconduct of a charity could impair individuals’ impression of the charity sector itself. More importantly, fraud victims from social out-groups tend to generalize blame and avoid other similar institutions and organizations [27]. Therefore, misconduct of a charity would not only impair individuals’ impressions of the tainted charity but also the donating behaviors through other charity sectors.

The negative effect of charity misconduct could be further amplified through mass media. The mass media’s effects on the general populace are wide-ranging and profound [15]. These could span the most minute things, such as personal decisions, to societal trends, such as suicide and crime rates [15,28,29,30,31]. The cultivation theory suggests that media contributes to people’s conceptions of social reality [32]. Negative information is a favorite of mass media and has a greater influence on individuals than positive information [33,34,35]. Humans tend to give greater weight to negative events, objects, or personal traits [35]. Negative media exposure has a negative impact on us [36,37,38,39], and our negativity bias makes us more vulnerable to negative events [35,39]. Previous studies found that media with prosocial and antisocial content influences individuals’ prosocial behaviors differently. Participants who played a prosocial or cooperative video game had more prosocial behaviors [40,41,42,43,44]. But when people were exposed to an antisocial newscast, they chose to cooperate in a later game less often than those who were exposed to a news broadcast containing prosocial content [45]. Similarly, individuals who watched positive social news (helping others) increased cooperation in the prisoner’s dilemma game and those who watched negative social news (bullying, child abuse, and dishonest behaviors) cheated more [46]. By impairing social trust, negative energy news reduced people’s helping behaviors [47]. Therefore, through the mass media, charity misconduct would be broadly spread, and knowing its effect would be crucial for coming up with coping strategies.

Not only the content of the news would influence individuals’ prosocial judgment and decisions [48], but also the presenting order. News is usually shown in a mixed manner instead of being presented alone. The order of presenting news has an impact on individuals’ emotions and cognition [49,50]. Concerning the preference of hearing the good news or the bad news first, news-givers prefer to deliver good news first, but news-receivers prefer to learn bad news first to reduce worry [49], showing the discrepancy between the preferences of news-givers and news-receivers. Presenting news in a certain order also influences recipients’ process of the news. An experience consisting of both positive and negative events can be evaluated as more satisfactory if the positive one occurs last [51]. Besides, the contrast model suggests that comparing the second information to the first one draws more attention to the second [52]. According to these previous findings, in the case of two sequentially presented pieces of news, if the first item is about news on charity misconduct while the second one is about a charity that carried out its duty, it might make the second news item more salient and leave a more positive impression. It would be a possible strategy to influence news receivers’ subsequential decisions or behaviors through manipulating the presenting order of news in order to align with their preference, that is, presenting charity nonmisconduct news (i.e., positive news) after charity misconduct news (i.e., negative news). It might draw more attention to the positive side and have a positive impact.

**Figure 1:**
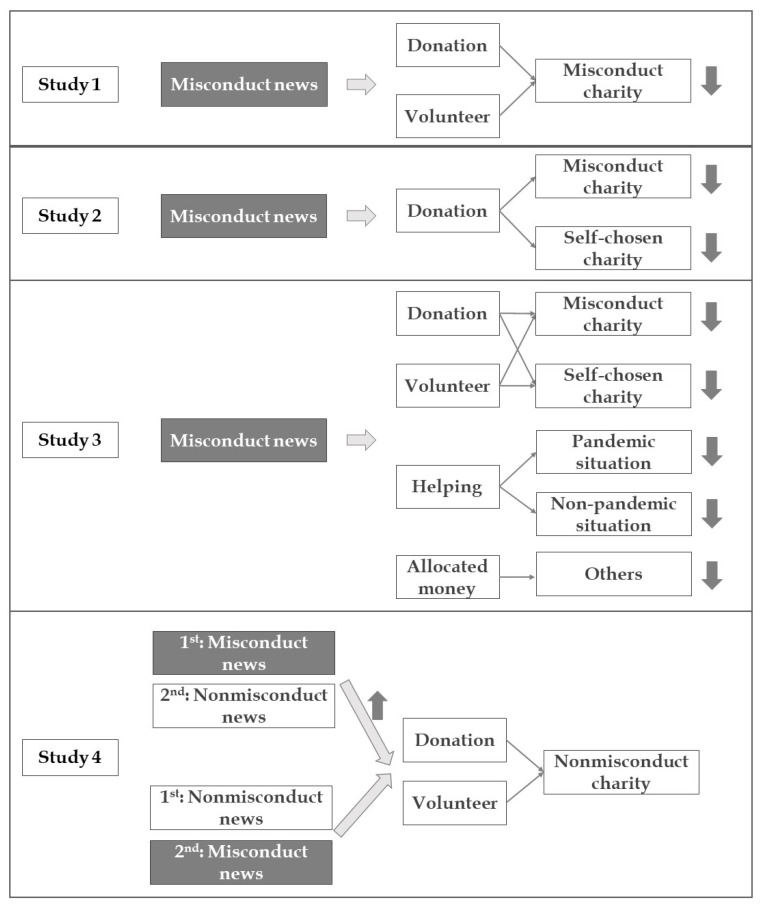
A summary diagram of Study 1 to Study 4. Participants read news on charity misconduct (dark) and news on charity nonmisconduct and reported their willingness to help in different conditions.

To investigate the impact of charity misconduct news of public health issues on individuals’ willingness to help and check if the manipulation of news presenting order to align with news receivers’ preference would increase their willingness to help, four studies were conducted. Figure 1 provides an overview of four studies. Study 1 investigated if the hypothetical news on charity misconduct would reduce participants’ willingness to donate to or volunteer in the reported charity by applying a within-subject design. Participants read two types of news: news about a charity receiving donations and supporting the work of fighting against the pandemic outbreak (charity nonmisconduct news); and news about a charity misappropriated donation (charity misconduct news). Since monetary donations and volunteer labor supply are two major types of philanthropic behaviors [53], individuals’ willingness on these two items in the pandemic context were assessed as the indexes reflecting their willingness to help in Study 1. Study 2 applied a between-subject design to find out if misconduct news would impair individuals’ willingness to donate to self-chosen charity sectors. The expectation was that participants would be less willing to offer help not only via the misconduct charity but also via other charity sectors after reading the nonmisconduct news. To further explore if the impairment of individuals’ willingness to help extends to non-pandemic related contexts, in Study 3 participants read two types of news and reported their willingness to help in different contexts. Study 4 explored a potential way to reduce the negative effect brought by charity misconduct by presenting charity nonmisconduct and misconduct news in a different order.

## 2. Study 1

Study 1 was conducted to find out if reading news about charity misconduct on public health issues would impair individual willingness to offer help via the reported charity. Two types of news (misconduct and nonmisconduct charity news) were presented to participants who later rated their willingness to help through the reported charity.

### 2.1. Methods

In Study 1, 281 valid questionnaires were collected through the Tencent Questionnaire platform. Targeted participants of this study were Chinese adults and were randomly recruited via convenient online postings on popular social media (WeChat). Interested participants returned their informed consent and completed the questionnaires. Data from 15 of them were excluded due to failing the manipulation-check questions (i.e., “Did the reported charitable organization work as they promised to the public?”) and attention-check questions (i.e., participants were instructed to respond as required). The remaining 266 participants (161 females, *M* = 22.70, *SD* = 4.71, 18 to 63) read two pieces of news in a row (see Supplementary Material). Among them, 224 (84.2%) are students, 180 (67.67%) had Bachelor’s degrees and 58 (21.80%) had Master’s degrees. On a 10-point Cantril scale of socioeconomic status [54], 70 (26.32%) reported that they were from middle socioeconomic status (6). A previous study about the effect of negative energy news on helping behaviors presented online news stories to participants and asked for their willingness to help [47]. A similar experimental design has been adopted in Study 1. Two sets of news that differ in ways of spending donations (i.e., carrying out their duty or not) were presented to participants. To avoid potential negative influence on real-life situations, before the presentation of the news participants were told that the following news were hypothetical (this manipulation was applied to all four studies). Every set had two types of news. In one set of news, one of the news items is about a charity that used donations to get private profits (charity misconduct news). The other one is news about a charity that used donations to help the people in need (charity nonmisconduct news). The presenting orders of the news were counterbalanced. After participants finished reading two pieces of news, they provided willingness ratings toward questions of interest: (1) donating tendency to the reported charity A or B: “If you have some extra cash, how much are you willing to donate the money to the organization A or B described in the news”; (2) volunteering tendency to the reported charity A or B: “If you have spare time and the charity A or B is recruiting volunteers who can work from home, how much are you willing to apply” on a 100-point scale (1 = very much unwilling to 100 = very much willing to).

### 2.2. Results

**Figure 2:**
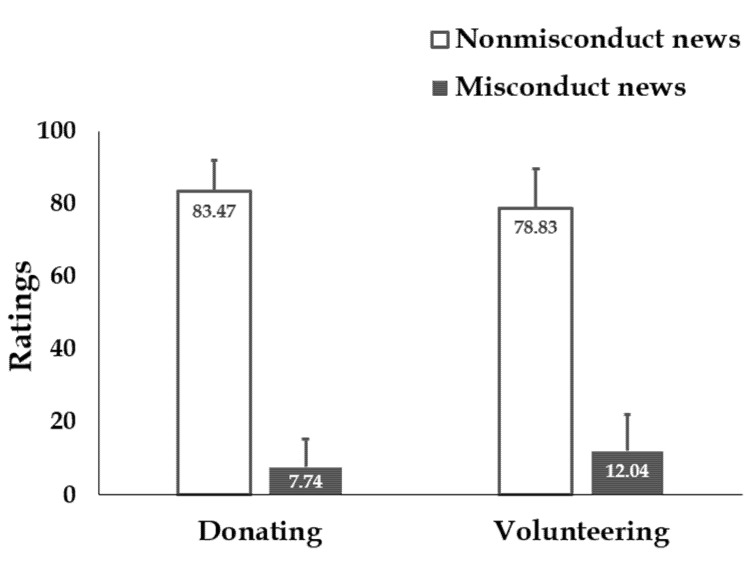
Results in Study 1. Participants’ willingness to donate to and volunteer at the misconduct charity was significantly lower than the nonmisconduct charity. Error bars: SD.

Results showed that participants’ willingness to donate to the misconduct charity (*M* = 7.74, *SD* = 15.16) is significantly lower than that to the nonmisconduct charity (*M* = 83.47, *SD* = 17.07; *t* (265) = 51.17, *p* < 0.001, *d* = 4.69; Figure 2). Similarly, participants’ willingness to apply to volunteer for the misconduct charity is significantly lower (*M* = 12.04, *SD* = 20.38) than that in the nonmisconduct charity condition (*M* = 78.83, *SD* = 21.69; *t* (265) = 36.13, *p* < 0.001, *d* = 3.14). Results showed that news on charity misconduct substantially decreased participants’ willingness to provide help via the misconduct charity.

### 2.3. Discussion

Through showing the news on the misconduct and nonmisconduct charity to participants, their willingness to donate to and volunteer at the reported charitable organizations was assessed. Results showed participants’ willingness to offer help via the misconduct charity was reduced in both aiding ways. The results confirm previous findings that donors are sensitive to aid impact and the effectiveness of charitable organizations [2,11].

However, in Study 1 participants read two types of news in a row and the differences observed between the misconduct and nonmisconduct conditions might be due to demand characteristics, that is, participants were aware of what the experimenters were expecting and how they were expected to behave, especially when two different types of news were presented to them. Besides, participants’ willingness to offer help via the assigned charitable organization (i.e., the reported charity) might be generally low and the impact of news on helping willingness might be moderated by their volitional choice of charity.

## 3. Study 2

Study 1 showed that participants were less willing to provide help via the misconduct charity. Fraud victims generalize blame to other similar institutions and organizations [27]. To find out if the negative effect of misconduct news on public health issues would be generalized to other charity sectors, participants’ willingness to donate to a self-chosen charity was investigated in Study 2. In the study, participants were randomly assigned to two groups, each of which was presented with only one type of news and reported their willingness to donate.

### 3.1. Methods

In Study 2, 505 questionnaires were collected through the Tencent Questionnaire platform, and 65 were excluded for failing the manipulation-check questions (i.e., “Did the reported charitable organization work as they promised to the public?”) and attention-check questions (i.e., participants were instructed to respond as required). The remaining 440 Chinese participants were randomly assigned to two groups: the nonmisconduct charity group (*n* = 218, 162 females, *M* = 22.84, *SD* = 5.229, range: 18–59) and the misconduct charity group (*n* = 222, 171 females, *M* = 23.11, *SD* = 5.852, range: 18–55). Among them, 343 (78%) were students, 314 (71.4%) had Bachelor’s degrees and 76 (17.3%) had Master’s degrees. On a 10-point Cantril scale of socioeconomic status, 121 (27.5%) reported that they were from middle socioeconomic status (6). Participants in the nonmisconduct charity group read a piece of news about a nonmisconduct charity (see Supplementary Materials) and then reported their willingness to donate to the reported charity and a self-chosen charity (“If you have some extra cash and you can choose any charitable organization you want, how much are you willing to donate”). Participants in the misconduct charity group read a piece of news on a misconduct charity and then reported their willingness to donate.

### 3.2. Results

**Figure 3:**
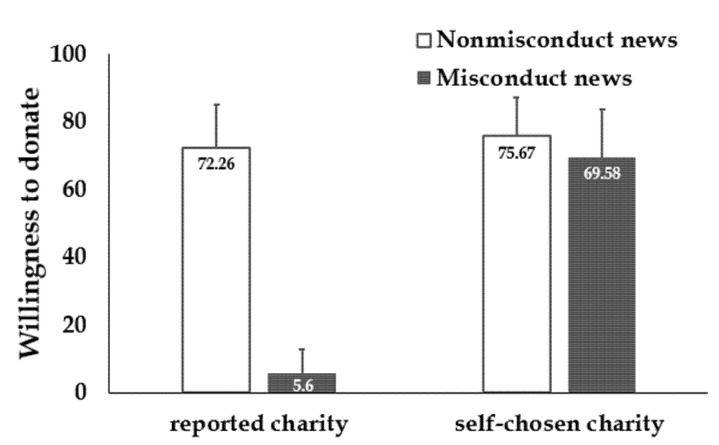
Results in Study 2. Willingness to donate to the reported charity and a self-chosen charity is significantly lower in participants who read charity misconduct news than those who read charity nonmisconduct news. Error bars: SD.

Results showed significant differences of willingness to donate to the reported charity between the two groups (*t* (337.52) = 33.93, *p* < 0.001, *d* = 3.26; nonmisconduct charity: *M* = 72.26, *SD* = 25.42; misconduct charity: *M* = 5.60, *SD* = 14.09; Figure 3). More importantly, even in the situation where they could choose any charity to donate, participants’ willingness to donate was significantly lower in the charity misconduct condition (*M* = 69.58, *SD* = 28.41) than that in the charity nonmisconduct condition (*M* = 75.67, *SD* = 23.11; *t* (423.41) = 2.47, *p* = 0.014, *d* = 0.24). Charity misconduct news impaired individual willingness to provide help via both the misconduct charity and any charity they preferred.

### 3.3. Discussion

Study 2 applied a between-subject design and allowed participants to donate to any charitable organization they preferred. The results still showed that their willingness to donate decreased after reading the misconduct news. Study 2 excluded the possible confounding explanation in Study 1 about demanding characteristics and hence supported that the negative impact on helping willingness is not caused by participants’ awareness of experimenters’ expectations. More importantly, when participants could choose any charitable organizations they prefer, their willingness to offer help was still damaged in the misconduct news condition, suggesting the negative impact is not caused by the lack of volitional choices of charity. However, the negative impact found in Study 2 was still limited to charity-related situations. Less is known if the negative impact could generalize to some other circumstances.

## 4. Study 3

Study 2 showed that the negative impact brought by misconduct charity news is not restricted to the misconduct charity per se. Reduced willingness to donate was found even when individuals could choose any charity they like. However, little is known as to whether it would also impair an individual’s willingness to offer help in some other situations that do not relate to charitable organizations or the pandemic outbreak context. Studies 1 and 2 focused on participants’ willingness to donate to or volunteer in pandemic contexts. In Study 3, participants were asked to rate their willingness to offer help in pandemic situations, non-pandemic situations where help was not provided via charity, and their other-regarding tendency in the dictator game (i.e., a commonly used economic game [19,55]). Through comparing ratings between two conditions where the misconduct and nonmisconduct news were presented, the impact of charity misconduct news on individuals’ general helping tendencies was investigated.

### 4.1. Methods

In Study 3, 313 questionnaires were collected and 24 of them were excluded due to failing the manipulation-check questions (i.e., “Did the reported charitable organization work as they promised to the public?”) and attention-check questions (i.e., participants were instructed to respond as required), leaving 289 valid questionnaires (209 females, *M* = 23.06, *SD* = 4.73, range: 18 to 50). Among them, 214 (74.0%) were students, 184 (63.7%) had Bachelor’s degrees and 76 (26.3%) had Master’s degrees. On a 10-point Cantril scale of socioeconomic status, 59 (20.4%) reported that they were from middle socioeconomic status (6). Participants read two pieces of news as Study 2 (i.e., a charity launched donation to support the fight against pandemic). The presenting orders of the news were counterbalanced. Every piece of news was followed by questions of willingness to provide six types of help: (1) willingness to donate to the reported charity; (2) willingness to volunteer at the reported charity; (3) willingness to donate to a self-chosen charity; (4) willingness to volunteer at a self-chosen charity; (5) willingness to help in the pandemic situation (providing help to support the fight against pandemic); (6) willingness to help in the non-pandemic situation (volunteer work like helping the elderly, or donating to poor people or families in need). In addition, to find out if the news changes people’s other-regarding tendency in a completely non-related situation, participants were asked to play a dictator game where they divided 100 yuan between themselves and a stranger.

### 4.2. Results

**Figure 4:**
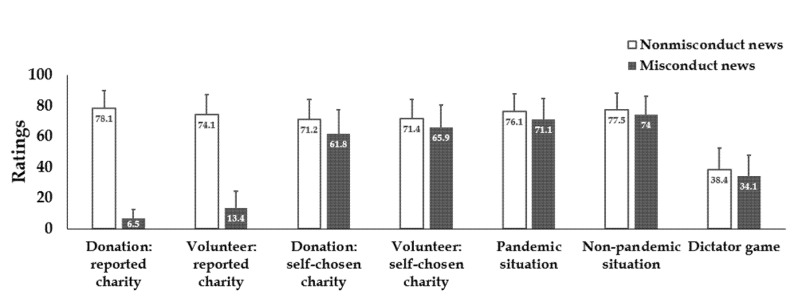
Results in Study 3. Reading news on charity misconduct significantly decreased participants’ willingness to provide help: donating to the reported charity, volunteering in the reported charity, donating to a self-chosen charity, volunteering at a self-chosen charity, helping in a pandemic situation, and a non-pandemic situation. Participants allocated less money to the other partner in a dictator game. Error bars: SD.

Results showed that news on charity misconduct significantly decreased participants’ willingness to provide help in six different ways (Figure 4; Table 1), including donating to the reported charity, volunteering in the reported charity, donating to a self-chosen charity, volunteering at a self-chosen charity, and helping in a pandemic situation and a non-pandemic situation. Even in the dictator game, reading news on the misconduct charity decreased participants’ allocated money to the other partner.

### 4.3. Discussion

Studies 1–3 showed that the negative impact of misconduct news consistently existed in various conditions, including pandemic situations, non-pandemic situations, and the dictator game. Even though misconduct news harmed individuals’ willingness to help in situations that are more alike to the reported circumstance in the news to a higher extent than those that are less alike, our findings still showed that general helping willingness and other-regarding tendency were damaged. Given that the negative impact of misconduct news could spread to irrelevant charitable organizations and situations, possible ways of reducing this impact should be explored.

## 5. Study 4

Study 4 explores a potential way to reduce the negative effect brought by charity misconduct. Presenting news in a certain order influences recipients’ process of the news and decisions [49,50,51]. To find out that if presenting news in different orders would influence participants’ willingness to provide help via the nonmisconduct charity, in Study 4 participants read two types of news in opposite orders. When nonmisconduct news was presented after misconduct news, individuals’ willingness to help via the nonmisconduct charity was expected to be enhanced. Furthermore, to check if the enhancement effect is brought by having a bad alternative (i.e., misconduct charity), in Study 4, after participants finished reading the news and reported their willingness ratings, they were asked to report their willingness to provide help toward the originally reported charity again. If merely providing a bad alternative could help enhance helping willingness, higher ratings should be observed. Otherwise higher willingness to offer help should be only observed in the condition when nonmisconduct news is presented after misconduct news.

### 5.1. Methods

In Study 4, 298 questionnaires in total were collected and 24 of them were excluded due to failing the manipulation-check questions (i.e., “Did the reported charitable organization work as they promised to the public?”) and attention-check questions (i.e., participants were instructed to respond as required). Among the remaining 274 participants, 209 (76.3%) were students, 170 (62.0%) had Bachelor’s degrees and 74 (27.0%) had Master’s degrees. On a 10-point Cantril scale of socioeconomic status, 83 (30.3%) reported that they were from middle socioeconomic status (6). The remaining participants were randomly assigned to two groups, non-mis group (*n* = 148, 99 females, *M* = 24.06, *SD* = 5.715, 18 to 52) and mis-nonmis group (*n* = 126, 95 females, *M* = 23.35, *SD* = 5.131, 18 to 55). In the non-mis group, participants read charity nonmisconduct news first, reported their willingness ratings about donation and volunteering, and then read charity misconduct news and reported willingness ratings. They also reported their willingness to help via the nonmisconduct charity again at the end. Participants in the mis-non group went through a similar procedure except that they read charity misconduct news first.

### 5.2. Results

**Figure 5:**
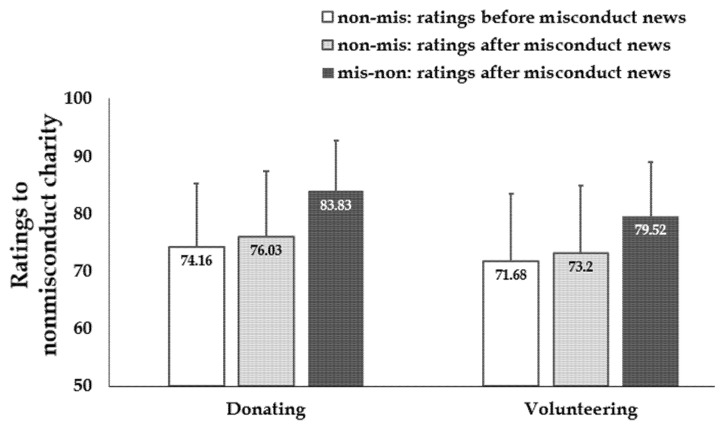
Results in Study 4. Donating and volunteering ratings to the nonmisconduct charity after reading misconduct news (mis-con condition) are significantly higher than ratings before reading misconduct news (non-mis condition) and ratings after reading misconduct news (non-mis condition). Error bars: SD.

Donation ratings toward the nonmisconduct charity after reading misconduct news (mis-non condition; *M* = 83.83, *SD* = 17.60) are significantly higher than ratings before reading misconduct news (non-mis condition; *M* = 74.16, *SD* = 22.28; *t* (270.55) = 4.011, *p* < 0.001, *d* = 0.48) and ratings after reading misconduct news (non-mis condition; *M* = 76.03, *SD* = 22.82; *t* (269.50) = 3.19, *p* = 0.002, *d* = 0.38; Figure 5). The results remain similarly with regards to volunteering ratings. Volunteering ratings toward the nonmisconduct charity after reading misconduct news (mis-non condition; *M* = 79.52, *SD* = 18.88) are significantly higher than ratings before reading misconduct news (non-mis condition; *M* = 71.68, *SD* = 23.39; *t* (271.26) = 3.07, *p* = 0.002, *d* = 0.37) and ratings after reading misconduct news (non-mis condition; *M* = 73.2, *SD* = 23.45; *t* (271.19) = 2.47, *p* = 0.014, *d* = 0.29).

### 5.3. Discussion

The results showed that higher willingness ratings were only observed in the condition where the nonmisconduct news is presented after misconduct news. After participants finished reading the news and reported their willingness ratings, they were asked to report their willingness to help toward the initially reported charity again, and higher ratings were not detected. The results suggest that the enhancement in helping willingness is not caused by merely having a bad alternative but by having a bad reference ahead.

## 6. General Discussion

Public health represents organized efforts and public support to prevent disease, prolong life and promote the health of the entire population [56]. As is often the case, public health seeks public support and sets up mechanisms for social mobilization [57]. Especially when we are facing public health crises like the COVID-19 pandemic outbreak, there are increasing demands on aid from public support and nonprofit sectors. But studies found a disproportionately high incidence of nonprofit fraud in the health and human services charitable sectors [58] that would potentially impair the effectiveness in the fight against public health crises. Besides, media attention has been always focused on cases of fraud [59]. On one hand, reporting nonprofit frauds to the public could help prevent fraud and avoid inefficient donations. On the other hand, people are sensitive to information about charitable organizations [6] and prefer charities that have a guaranteed impact over those that have a less certain impact [60]. Little is known about how news reports on charity misconduct influences individuals’ willingness to help via the reported charity, unnamed charity, and their general helping willingness. The current study investigated if the news on charity misconduct reduces individual willingness to offer help as a whole and explored the potential ways to reduce negative impact. Through presenting news about charity misconduct and non-misconduct on public issues, our study found that news on charity misconduct impaired individuals’ general willingness to provide help (Figure 1). Study 1 showed that news on a misconduct charity substantially reduced individuals’ willingness to donate to or volunteer in the misconduct charity. Since individuals are more likely to donate money to their favorite charity [61], willingness to donate to any charity that participants prefer was also assessed in Study 2 and we still observed the negative impact brought by charity misconduct. Even when participants were asked to provide help in charity and pandemic irrelated contexts (Study 3), willingness to provide help was still reduced when they read the misconduct news, suggesting that the negative impact of misconduct news on willingness to help generalizes to a wider extent.

Prosocial behaviors are vulnerable to negative information [2]. Prosocial outcomes can be decreased by violent video games [62], and prosocial material could increase one’s interpersonal empathy and prosocial tendencies [62,63]. Even when individuals encountered unfair treatments, their subsequent charitable giving to an innocent third party was reduced, showing a generalized trend [64]. In our study, people’s willingness to help via charity is harmed by news on charity misconduct and the damage was generalized to the situations that have little to do with the circumstances described in the news. According to social learning theory, people learn by observation [65] and prosocial behaviors are a product of social learning [66,67]. A meta-analysis study found that the estimate of the prosocial modeling effect is medium [67]. Even though participants did not directly witness prosocial behaviors in our study, reading news about charity misconduct did provide them additional information about the effectiveness of donating behaviors and the quality of charities or projects. The image and reputation of charitable organizations exert a strong influence on donors’ behaviors [68], and cumulative instances of reporting on nonprofit misconduct would bring damage to the reputation of charitable sectors [58]. Donors care about the efficacy and trustworthiness of a charity and would search for information to confirm that their donation will be used properly [12]. A systematic literature review proposed that the perceived quality of charities or projects is one of the most important mediators that would influence the impact of social information on charitable giving [69]. The exposure to misconduct news might cause doubt in providing help via charity sectors and increase the sense of risky donations. In particular, individuals act more averse to charity risk than self-risk [70]. Perceived risky donations might further decrease people’s general helping willingness.

Previous studies investigated strategies that could increase charitable giving. For example, requesting additional importance ratings for charities increased donations and bequest intentions for similar unnamed charities [71]. Besides, providing a list of default charities would also increase donors [72]. In Study 4, we explored ways that could increase willingness to help and found that presenting charity misconduct news before nonmisconduct news increased participants’ willingness to provide help via the nonmisconduct charity. People prefer to encounter bad things first, but then experience a happy ending [51,73]. Presenting misconduct news before nonmisconduct news aligns with news receivers’ preference. The alignment between presenting ways and receivers’ preference rather than having a bad alternative might be the cause of individuals’ attitude change. Furthermore, individuals tend to use prior information as a reference point for behavioral adjustments [69]. The initially presented news might function as a reference point. Charity misconduct makes the following non-misconduct charity a better choice that could make them think their donation will be used properly. Our study suggests a simple manipulation in presenting news order that could reduce the negative impact of misconduct news on unnamed charities. The result highlights the importance of the presenting order of news.

Misconduct of charitable organizations has become more evident to the public and draws increasing attention with the help of the media. The media have a great impact on our ways of thinking and acting, but little was known about how news on misconduct charities would influence our virtue of wanting to help others. Our findings show how news on charity misconduct could influence individuals’ willingness to provide help. A better understanding of the impact of charity misconduct news could yield valuable insights and applications in real life. The findings provide implications for charitable organizations: (1) charitable organizations should prevent potential occupational fraud and convince the public that they are capable to ensure that resources are being properly managed and deployed; and (2) charitable organizations should be alert to news of misconduct in other malpracticing charitable organizations and prepare for a potential decline of donations. Moreover, the findings provide important implications for the media in that there is a potential positive impact on charitable organizations with good reputations that can be realized by manipulating the presentation order of news. Reporting positive news after negative news might help people gain confidence about the reality and take helpful actions in improving the situation.

## 7. Conclusions

The current study investigated if charity misconduct news reduced individuals’ willingness to offer help as a whole and explored potential ways to reduce the negative impact. Charity misconduct on public health issues significantly reduced participants’ willingness to offer help through donating or volunteering to not only the misconduct charity but also to a self-chosen charity, in the charity and pandemic associated context, in the charity and pandemic unrelated context, and even reduced other-regarding tendency in the dictator game. Despite the finding that news on charity misconduct impairs people’s willingness to help in general, presenting the nonmisconduct news after the misconduct news helps increase individuals’ willingness to provide help via the nonmisconduct charity. Our findings shed light on the negative impact of charity misconduct news on public health issues, suggest a potential way to nudge people to provide help, and provide implications for charitable organizations and the mass media.

## 8. Limitations

Our study has a limitation. Participants’ willingness to offer help was measured by a single item, which might reduce internal validity and reliability [74]. Despite the fact that we replicated the results that charity misconduct news reduced willingness to help in four studies, there might be a discrepancy between individuals’ expressed willingness and actual donating behaviors. Field experiments are a major tool for investigating helping and charitable giving [75,76,77], and should be considered in future studies to investigate the impact of charity misconduct news about public health issues on prosocial behaviors.

## Figures and Tables

**Table 1:** Results in Study 3.

Helping Types	Charity Nonmisconduct	Charity Misconduct	*t*	*d*
	Mean (s.d.)	Mean (s.d.)		
Donating to the reported charity	78.08 (22.97)	6.45 (12.42)	46.63 ***	3.88
Volunteering in the reported charity	74.10 (25.95)	13.36 (21.68)	32.95 ***	2.54
Donating to a self-chosen charity	71.19 (25.55)	61.84 (30.62)	6.21 ***	0.33
Volunteering at a self-chosen charity	71.42 (25.54)	65.91 (28.77)	4.24 ***	0.2
Helping in the pandemic situation	76.09 (23.04)	71.10 (27.07)	4.04 ***	0.2
Helping in the non-pandemic situation	77.49 (21.63)	73.98 (24.20)	4.45 ***	0.15
Allocated money to others in the dictator game	38.42 (28.29)	34.13 (27.76)	6.19 ***	0.15

Note: * *p* < 0.05; ** *p* < 0.01; *** *p* < 0.001.

## Supplementary Materials

The following are available online at https://www.mdpi.com/article/10.3390/ijerph182413039/s1, file S1: The materials used in Study 1 and 2.
