# Supplementary material for: Charity Misconduct on Public Health Issues Impairs Willingness to Offer Help
Source: Int J Environ Res Public Health. 2021 Dec 10;18(24):13039. doi: 10.3390/ijerph182413039 (PMC8700860; doi:10.3390/ijerph182413039)
Supplement: Supplementary file 1 [file ijerph-18-13039-s001.zip › ijerph-1450925-supplementary.pdf]

Supplementary Materials: The materials used in Study 1 and 2 are listed as below. All materials are fictitious.

Study 1:

News on charity nonmisconduct (version 1): "To support the work of fighting against the pandemic outbreak. A charitable organization A received donations to purchase equipment and materials to produce surgical masks and protective clothing. They promised to use the product to donate to hospitals that were assigned to treat patients. Up until now, the organization has produced a batch of surgical masks and protective clothing. The products have been delivered to the local hospitals and the details of the funds have been made public. The distribution of the products has been reported in public every day and the local authorities confirmed that the content is true and accurate."

News on charity misconduct (version 1): "To support the work of fighting against the pandemic outbreak. A charitable organization A received donations to purchase equipment and materials to produce surgical masks and protective clothing. They promised to use the product to donate to hospitals that were assigned to treat patients. Up until now, the organization has produced a batch of surgical masks and protective clothing, but it has not yet made the donation procedure public. Later investigations have shown that the products have been diverted and resold. The products were not donated to the hospitals."

News on charity nonmisconduct (version 2): "After the pandemic outbreak, a charitable organization B launched aid operations to support the work of fighting against the pandemic outbreak. Because the major pandemic areas are lack of food and supplies, the organization promised to use the donation to purchase food and supplies and donate them to the elderly and families with financial difficulties who live in the major pandemic areas. Up until now, the organization received a lot of donations and used the money to get food and supplies. It later donated them to the elderly and families with financial difficulties. The details of the funds have been made public and the local authorities confirmed that the content is true and accurate."

News on charity misconduct (version 2) "After the pandemic outbreak, a charitable organization B launched aid operations to support the work of fighting against the pandemic outbreak. Because the major pandemic areas are lack of food and supplies, the organization promised to use the donation to purchase food and supplies and donate them to the elderly and families with financial difficulties who live in the major pandemic areas. Up until now, the organization received a lot of donations and used the money to get food and supplies. But it has not made the distributions in public and no supplies have reached the associated areas. Later investigations have shown that the supplies have been diverted and resold."

Study 2:

The materials used in Study 2 are listed as follows:

News on charity nonmisconduct: "After the pandemic outbreak, charitable organizations across the country spontaneously launched aid operations for the hardest-hit area. Among them, a charitable organization A received donations, including money, surgical masks, medical gloves, protective clothing and food at the beginning of the outbreak. The money was supposed to use to purchase medical supplies and sent to the hardest-hit area and designated locations. Up until now, the donated materials have been delivered to the hardest-hit area and designated areas. The staff of the non-profit organizations confirmed with the local staff about the distribution of materials. Now the supplies have reached and the details of the funds have been made public. The distribution of the materials has been reported in public every day and the local authorities confirmed that the content is true and accurate."

News on charity misconduct: "After the pandemic outbreak, charitable organizations across the country spontaneously launched aid operations for the hardest-hit area. Among them, a charitable organization B received donations, including money for surgical masks, medical gloves, protective clothing and food at the beginning of the outbreak. The money was supposed to use to purchase medical supplies and sent to the hardest-hit area and designated locations. Up until now, none of the supplies from that organization has reached the hardest-hit area or other designated locations. Donors and the public have requested them to announce the donations, but the organization has not made it public. Later investigations have shown that the donations have been diverted and resold."
